# A Novel Technique Combining Human Acellular Dermal Matrix (HADM) and Enriched Platelet Therapy (EPT) for the Treatment of Vaginal Laxity: A Single-Arm, Observational Study

**DOI:** 10.1007/s00266-022-02805-x

**Published:** 2022-02-23

**Authors:** Fang Yang, Yin Liu, Hong Xiao, Jiaying Ma, Huanying Cun, Chengdao Wu

**Affiliations:** 1 Department of Dermatology, Aesthetic Gynaecology and Plastic surgery, Yunnan Wushi Jiamei cosmetic Hospital, No.300, Dongsi street, Kunming, 650000 Yunnan Province China; 2 Department of Plastic Surgery, The 2nd Hospital affiliated to Kunming Medical University, No.374, Dianmian street, Kunming, 650000 Yunnan Province China

**Keywords:** Vaginal laxity, HADM, EPT, Surgical

## Abstract

**Background:**

There is a paucity of knowledge about cosmetic vaginal tightening procedures; therefore, the present study aimed to describe the clinical effects of a novel combination technique of human acellular dermal matrix (HADM) and enriched platelet therapy (EPT) for the treatment of vaginal laxity.

**Methods:**

This single-arm, observational study was conducted on 52 patients with grade II to III vaginal relaxation. HADM biological band (U-shaped) was implanted in these patients by submucosal puncture in vagina under anesthesia. This was followed by thrice administration of EPT injection, once at the time surgery followed by each dose at a time interval of one month. Patients were followed up for a period of 6 months based on Female Sexual Function Index (FSFI) and Vaginal Health Index (VHI) scores. Patient satisfaction was measured using Visual Analogue Score (VAS).

**Results:**

About 52 women with median age of 39 years were included in the study. The average time reported to complete HADM surgery was reported as 27 minutes. Following implantation, it was found that labia minora was significantly closed and perineal length was increased from 1.5 to 2.2 cm. Moreover, there was improvement in elasticity, contractility and lubricity of vaginal mucosa. The sexual function scores from pre- to post-surgery were significantly increased (7.95 vs. 30.09; *p* value: <0.001). The mean VHI score also increased significantly after 6 months of treatment (mean ± S.D. before vs after treatment: 11.2 ± 3.3 vs. 19.6 ± 4.1, *P* < 0.0001). The mean VAS after surgery was 1.61 ± 0.31. About 96% of the patients did not feel any pain after treatment at 6-month follow-up. No adverse effects were reported in this study.

**Conclusions:**

These findings supported that combination treatment with HADM and EPT was safe and associated with both improved vaginal laxity and sexual function. These results may provide a novel surgical technique for this prevalent and undertreated condition.

**Level of Evidence IV:**

: Therapeutic Study This journal requires that authors assign a level of evidence to each article. For a full description of these Evidence-Based Medicine ratings, please refer to the Table of Contents or the online Instructions to Authors www.springer.com/00266.

## Introduction

Sexual dysfunction is a common complex multifactorial issue faced by many married women [1]. Vaginal laxity and dryness are highly prevalent conditions among women having a significant impact on a woman’s sexual health, body image and quality of life [1, 2]. Vaginal laxity or excessive vaginal looseness is often recognized as decreased sensation during sexual intercourse and believed to be underreported by about 80% of women [2]. Data collected from 2621 women revealed that vaginal laxity was correlated with symptoms of prolapse, stress urinary incontinence, overactive bladder, decreased vaginal sensation during intercourse, and poor sex life [2]. Vaginal laxity is known to create a gap in perineum and reduces frictional sensation leading to low sexual satisfaction [3]. Vaginal tightening procedures such as vaginoplasty and perineoplasty is mainly used for treatment of vaginal constriction [3]. A number of techniques have been described in the literature for labiaplasty namely, deepithelialized reduction, linear incision, composite reduction, wedge reduction, W-plasty excision or Z-plasty. Out of them, linear excision is most commonly preferred by majority of gynecologists owing to its simple and minimally invasive approach [4].

Human acellular dermal matrix (HADM), an extracellular matrix of three-dimensional cell scaffold structure had been used widely for wound repair, tissue regeneration and plastic surgery with good histocompatibility. The antigenic components of HADM that might cause immune rejections are generally removed by physical or chemical decellularization process. HADM is known for low absorption rate after implantation and inducing angiogenesis in host tissue. HADM is used for urethral reconstruction, congenital classic bladder exstrophy, penile skin defect and hidradenitis suppurativa [5]. Considering the above uses, HADM technique can prove to be an innovative and minimally invasive method and can be applied as biological band in vaginal constriction. In recent years, the common method mostly adopted by our surgical team is to implement HADM biological band vaginal constriction in U-shaped design [6, 7]. We have usually observed that in clinical practice, patients implementing HADM biological band tightening generally reported significant dryness especially within 3 months after surgery. On the other hand, the enriched platelet treatment (EPT) showed a remarkable nourishing effect on the vaginal mucosa by improving the secretion. Platelets release about 35 growth factors thereby promoting tissue healing and regeneration. Aesthetic gynecologists utilize this technique in vaginal rejuvenation and O-shot therapy. Rejuvenation of genitalia by these methods provided a pleasant appearance to the patient [8]. Previous studies on postmenopausal women for vulvovaginal atrophy showed that autologous platelet-rich plasma (PRP) combined with hyaluronic acid (HA) improved the trophicity and hydration of vaginal mucosa [9]. Also, PRP was a promising approach in promoting vaginal fibroblast attachment in patients with pelvic organ prolapse, demonstrating its role in urogynecology surgeries [10]. So far, no standard procedures are available to improve the trophic and dimensional alterations of the vaginal area [11]. Therefore, the present study aimed to describe the clinical effects of HADM biological band technique for the treatment of vaginal laxity. Moreover, the present study explored the effects of EPT plus HADM in improving the mucosal performance in vagina.

## Methods

### Study Design

This non-randomized, single-arm, observational study conducted between January 2021 and June 2021 enrolled 52 patients in the clinical observation group. Patients were included based on the following criteria: women aged ≥ 18 years with grade II~III vaginal relaxation, 3–6 months post-partum, physical examination must have vaginal cavity and mouth relaxed to varying degrees, flat vaginal mucosa, collapsed perineal area, and shortened linear distance between vagina and anus, inner diameter line of vagina (≥ 3 transverse fingers), loose lateral wall with weak vaginal contraction. Patients with menstruation, pregnancy, severe perineal laceration and contraindications were excluded. The study protocol was approved by Ethics Committee (Institutional Review Board) of Yunnan Wushi Jiamei Cosmetic Hospital, Yunnan, China (YWJC008956C) and conducted in accordance with the Declaration of Helsinki. Written informed consent was obtained from all the patients before participation in the study.

### Preoperative Preparation

Surgery was conducted from 3~5 days after the end of menstruation to 10 days before start of the next menstrual cycle. Moreover, patients must abstain from sexual activity for 3 days before surgery.

### HADM Biological Band Surgical Technique

Surgery was conducted under routine intravenous anesthesia so that patient can comfortably lie down in the bladder lithotomy position. Disinfect the perineum and vagina using single-use disposable towels impregnated with a disinfectant (0.5% iodophor). In order to increase the vaginal rectal space, 40 ml of swelling solution comprising 0.5% lidocaine (20 ml), ropivacaine and normal saline (10 ml each) and dexamethasone (10 mg) was injected into the lateral and posterior walls of vagina and perineum. HADM biological strap (1.0 cm×15cm) procured from Beijing Jayyalife Biotechnology Co. Ltd. was rinsed thrice with normal saline (0.9% sodium chloride solution) and set aside.

**Fig. 1:**
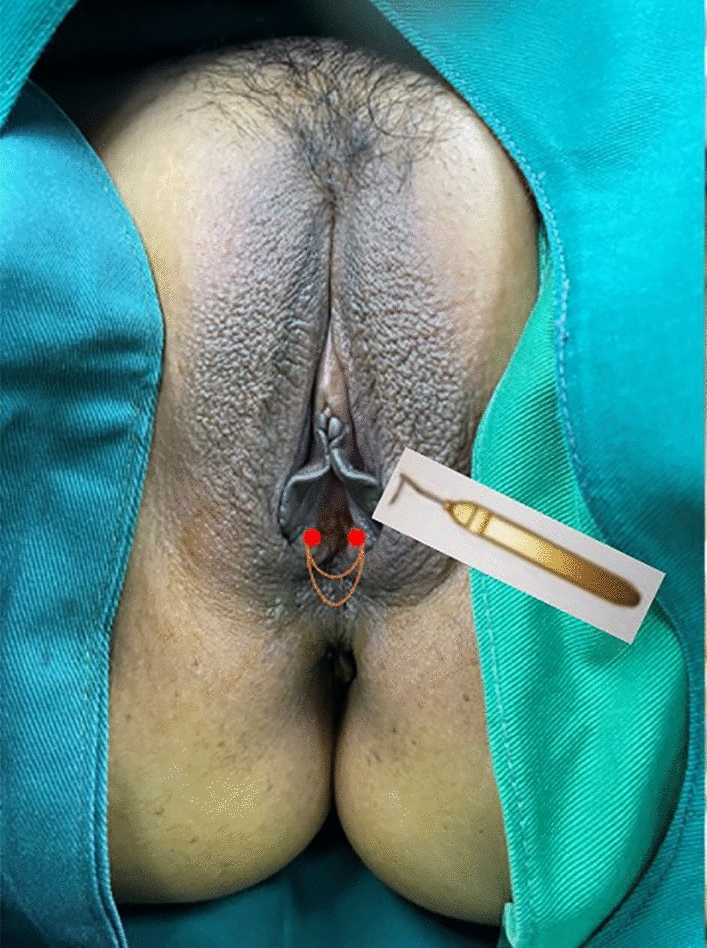
HADM U-shaped suture

About 0.5-cm-long incisions at 3-5 points and at 7-9 points were given inside and outside the hymen mark, respectively (Fig. 1). Submucosal puncture was given from the left side by right puncture guide, and needle exited from the right side from the submucosal muscle layer of the anterior and posterior wall of the vagina. The HADM strap was fixed on to the tail end of the silk thread so as to guide the strap into the puncture tunnel to complete a U-shaped suture. Similarly, using the left puncture guide needle, the silk thread from the right incision was sneaked through different layers in the submucosal muscle layer of the vaginal side and rear wall so that the biological strap exits out from the left incision to complete the "U" suture again. The biological strap was pulled and tightened at both ends to narrow the vaginal cavity so that about 1.5 fingers can be accommodated.

### Preparation of EPT Injection

**Fig. 2:**
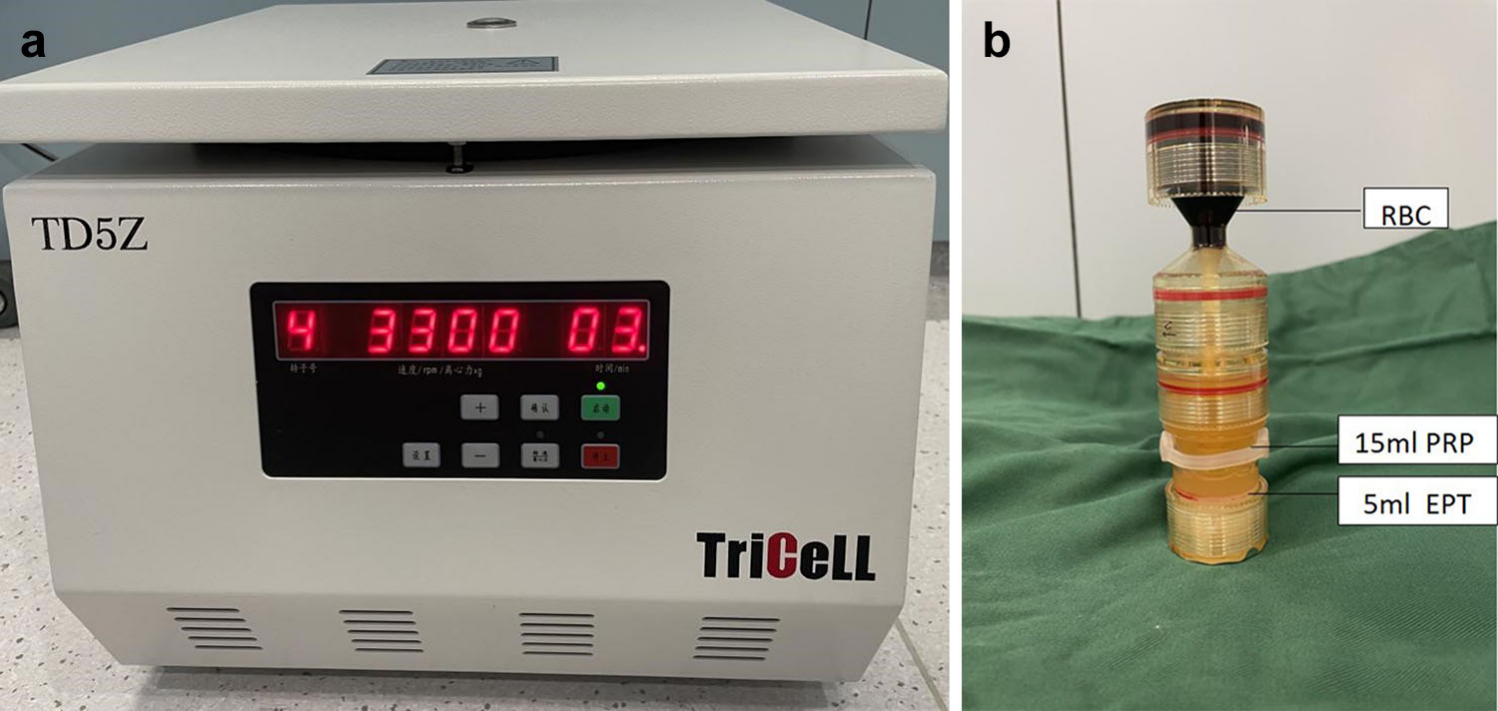
A Livsraft EPT and centrifugal equipment. B Livsraft centrifuge tube

Livsraft EPT (REV-MED Inc, Korea) and centrifugal equipment (Fig. 2A) procured from EVECHARM was used to prepare vaginal mucosal injection. Livsraft EPT has a matching preparation device, and the injection was prepared as follows: About 40 ml of blood sample was collected from the elbow vein and injected into the specific centrifuge tube of Livsraft. Blood sample was centrifuged twice, first at a speed of 3300 rpm for 4 minutes. Centrifuge tube was taken out, and red blood cells were sealed. The centrifugation process was repeated again at 3300 rpm for 3 minutes. Following this, it can be clearly observed that about 15 ml of clear light-yellow platelet-rich plasma (PRP) and 5ml of very high concentration platelet plasma to be used for EPT are separated in the centrifuge tube (Fig. 2B). These can be extracted by syringe from the bottom end of the centrifuge tube. Following extraction, platelet count was performed, revealing an average count of 248 × 10^9^/L. The average red blood cells, white blood cells, hemoglobin and hematocrit counts were 5.6 × 10^12^/L, 5.6 × 10^9^/L, 121g/L, and 42%, respectively. PRP was stored under normal light condition at 24–28 °C.

### Administration of EPT Injection

For every 40 ml of withdrawn blood, 15 ml of PRP (high concentration) and 5 ml of very high concentration PRP was obtained, which was injected using 30g sharp needle to surround multi-point injection into vaginal mucosa. Using the same needle, inject 2ml of EPT into point G, 1ml in point A area of point U and 1ml in clitoris. The G-spot is located at about 5cm away from urethral orifice, a bean-shaped region. The A point is located between G-spot and cervix. It can receive direct stimulation or can be stimulated by rubbing the vaginal wall, while U point is located at about 5 cm away from urethral orifice and the patient feels the urge to urinate on stimulation. Multiple studies have agreed on the existence of G-spot, but there was no agreement on the exact location, size, or nature [12]. However, lately, various literatures have rebutted the original concept of G-spot from evolutionary, anatomical, and functional points of view. So far, only few studies were able to describe the psychological, behavioral, and social correlates of the pleasure due to induction of G-spot or vaginally induced orgasm (VAO) [13]. After surgery, gauze was applied to compress the vaginal area for 10 minutes to cease bleeding. After 10 minutes, vaginal area was checked for no active bleeding points. The EPT therapy was given thrice post-surgery. The first injection was given during placement of biological band, followed by second and third injection at an interval of one month post-surgery.

### Follow-Up

The primary endpoint was to assess Female Sexual Function Index (FSFI) in patients when they were followed up for 3-6 months which included parameters such as female sexual function, vaginal tightness and vaginal moisture. The FSFI was validated by Rosen et.al for the assessment of key dimensions of female sexual function.[14] The evaluation of sexual function was aimed at six items, namely sexual arousal, orgasm, sexual satisfaction, sexual desire, vaginal wetting and sexual intercourse pain. The lowest score considered was 2 points, and the highest score was 36 points. Female sexual dysfunction (FSD) was reported as scores ≤ 26.55. Female with score of 26 and above was considered as having normal sexual function as noted first by Blümel et al. [15]. The secondary endpoints were to assess efficacy of the treatment based on the Gloria Bachmann Vaginal Health Index (VHI). The VHI evaluates 5 parameters (vaginal elasticity, vaginal secretions, pH, epithelial mucous membrane, vaginal hydration) and allows to obtain a final score defining the degree of atrophy in the genitourinary tract by assigning a single score to each parameter. Total score ranges from 5 to 25, with lower scores corresponding to greater urogenital atrophy. Patient satisfaction with the treatment was evaluated using a visual analog scale (VAS) at 6 months.

## Results

A total of 52 patients were included in this study with an age range of 21 to 52 years (median age: 39 years). A history of vaginal delivery (*n *= 35), cesarean section (*n *= 5), secondary delivery (*n *= 12), vaginal spontaneous delivery and cesarean section (*n *= 1 each) and abortion (*n* = 23) was reported. The average time reported to complete HADM biological band tightening surgery was 27 minutes, and the time from preparation to injection of EPT was about 30 minutes. Blood loss estimation was performed by gauze weighing method which revealed a loss of 5 and 0 ml after 24 hours of surgery with HADM and EPT injection, respectively. Following surgery, bleeding stopped after 3 days and < 24h with HADM and EPT injection, respectively.

**Fig. 3:**
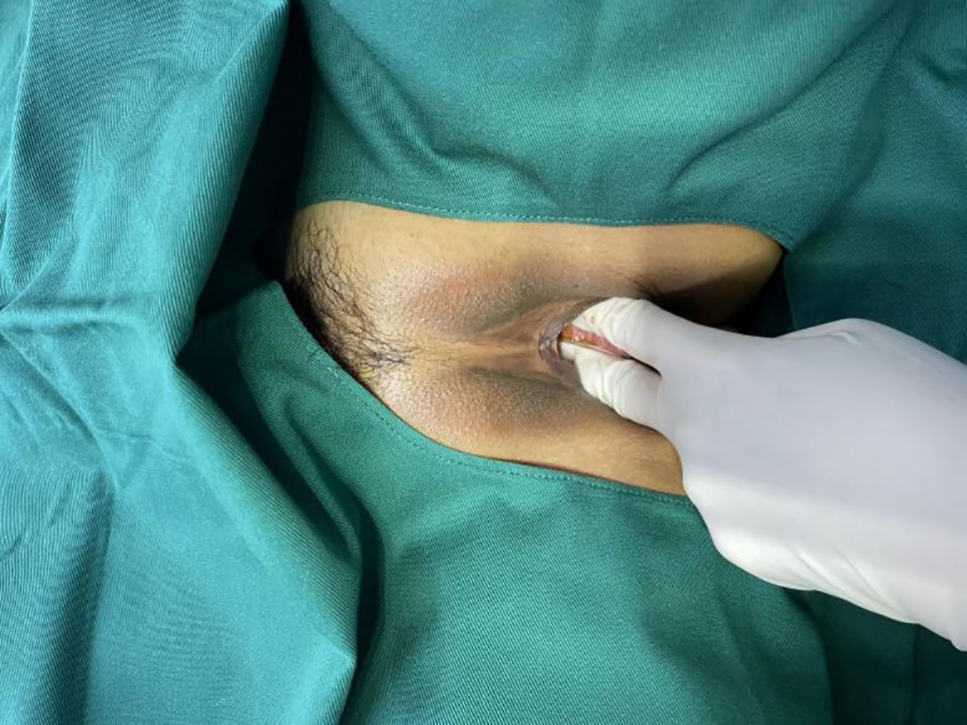
Vaginal cavity before HADM implantation

**Fig. 4:**
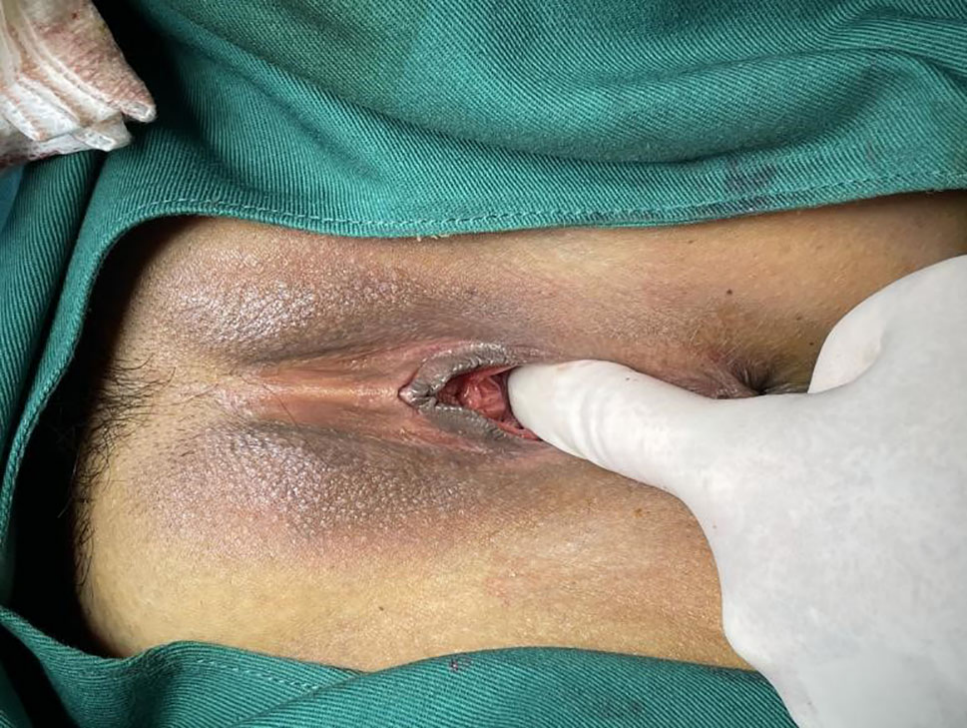
Vaginal cavity immediately after HADM implantation

**Fig. 5:**
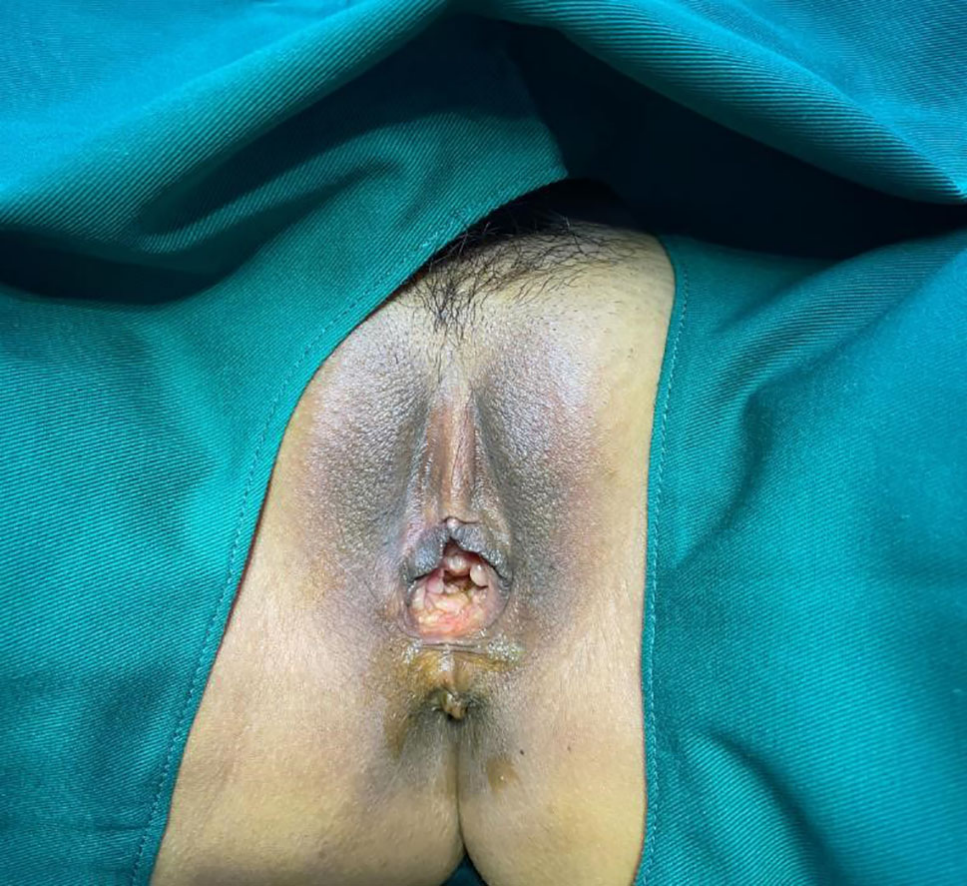
Dilated vulva mouth before HADM implantation

**Fig. 6:**
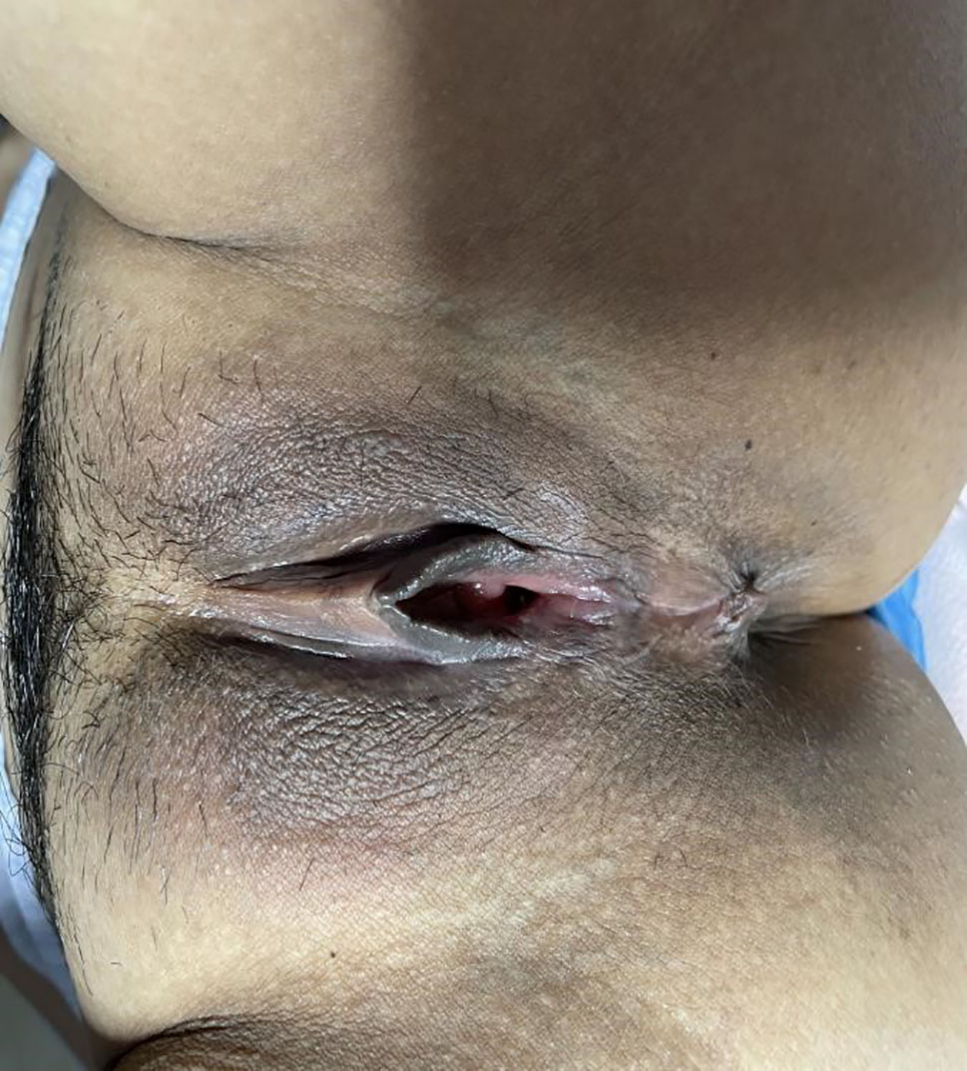
Vulva mouth after three months of HADM implantation

**Fig. 7:**
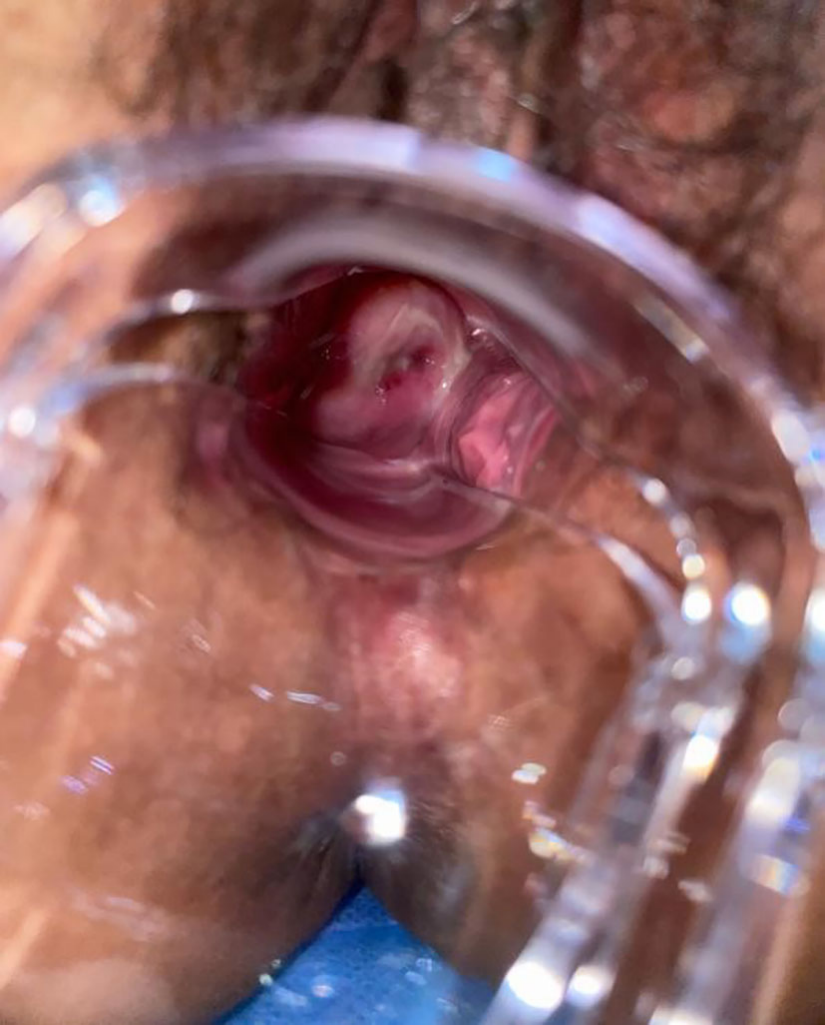
Flat vagina mucosal folds before EPT injection

**Fig. 8:**
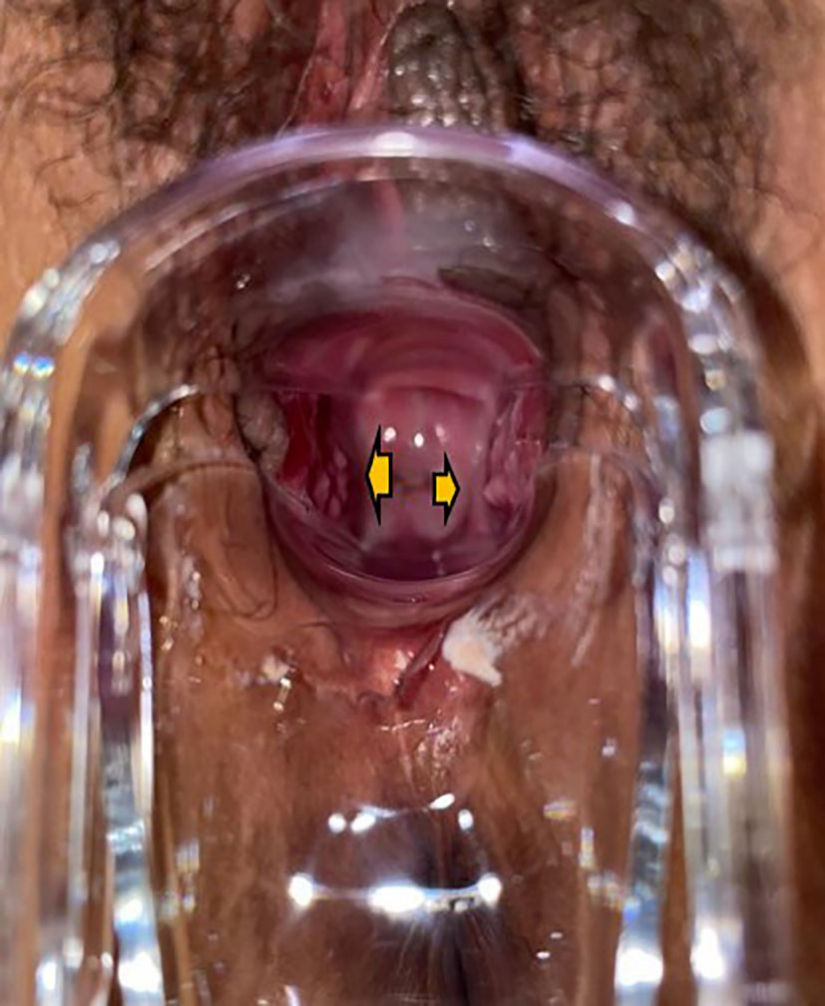
Vaginal mucosal folds after EPT injection

Following surgery, it was observed that the labia minora was significantly closed, perineum appeared fuller, and the linear distance between vagina and anus was significantly longer as compared to before surgery (average 0.5cm). The vagina was accommodating two transverse fingers before surgery (Fig. 3) which was reduced to one transverse finger post-surgery (Fig. 4). Prior to HADM implantation, the vulva mouth was dilated and the length of perineal body was 1.5 cm (Fig. 5). After 3 months, it was observed that size of the vulva mouth closure was reduced. Moreover, the perineal length was increased to 2.2 cm (Fig. 6). The active and passive elasticity of the vagina is enhanced, along with a significant increase in the contractility and tension of the vaginal walls. The folds of vaginal mucosa and water lubricity were increased significantly (Figs. 7 and 8), with no obvious adverse effects.

### Female Sexual Function Index Results

After 3–6 months of follow-up, the FSFI score was 94% which was found to be higher than the baseline. In addition, there was an outstanding improvement in the pre- and postoperative sexual function parameters score (7.95 versus 30.09; *p* value: <0.001) (Table 1).

**Table 1:** Sexual function parameters before and after HADM implantation surgery

Parameters	Before surgery	After surgery	*P* value
Sexual desire	1.2	4.80	0.013
Sexual arousal	1.09	4.80	0.003
Vaginal wetness	1.28	5.07	<0.001
Orgasm	1.40	5.08	<0.001
Sexual Satisfaction	0.94	5.06	<0.001
Sexual intercourse pain	2.04	5.28	0.018
Total score	7.95	30.09	<0.001

### Vaginal Health Index Results

The mean VHI score was increased significantly after 6 months of treatment when compared to before treatment (mean ± S.D. before vs. after treatment: 11.2 ± 3.3 vs. 19.6 ± 4.1, *P* < 0.0001). The elasticity and vaginal secretions had increased significantly at 6 months after treatment (mean ± S.D.: 2.35 ± 0.71 and 2.33 ± 0.75 before treatment, 3.68 ± 0.31 and 3.99 ± 0.51 after treatment, respectively) (*P *< 0.0001). There was a gradual decrease in the vaginal pH from baseline to 6 months post-surgery (6.5 ± 0.5 before treatment, 5.8 ± 0.56 at 1 month, 5.6 ± 0.11 at 3 months, 5.4 ± 0.28 at 6 months, respectively). The moisture rate increased by twofold compared to before treatment (2.5 ± 0.72 before treatment, 4.2 ± 071 at 6 months) (*P* < 0.0001). The epithelial integrity also increased at 6 months compared to before treatment (2.3 ± 1.12 before treatment, 3.9 ± 0.72 at 6 months) (*P* < 0.01) (Table 2).

**Table 2:** Vaginal Health Index before and after HADM implantation surgery

	Pre-treatment	1 month	3 months	6 months
Elasticity	2.35 ± 0.71	2.98 ± 0.31 (NS)	3.68 ± 0.31^a^	3.68 ± 0.31^a^
Fluid volume (pooling of secretion)	2.33 ±0.75	3.46 ± 0.66^a^	3.66 ± 0.51^a^	3.99 ± 0.51^a^
Ph	6.5 ± 0.5	5.8 ± 0.56^a^	5.6 ± 0.11^a^	5.4 ± 0.28^a^
Epithelial integrity	2.31± 1.12	3.0 ± 0.72 (NS)	3.8 ± 0.72 ^b^	3.9 ± 0.72 ^b^
Moisture	2.5 ± 0.72	3.6 ± 071^a^	4.1 ± 071^a^	4.2 ± 071^a^
Total	11.2 ± 3.3	15.7 ± 2.29^a^	19.11 ± 2.47^a^	19.6 ±14.1^a^

(Values in mean ±S.D.)

*NS* nonsignificant; *SD* standard deviation.

^a^*P* < 0.0001.

^b^*P* < 0.01.

### Patient Treatment Satisfaction

The mean on VAS after surgery was 1.61±0.31, indicating a good quality of patient satisfaction. About 96% of the patients did not feel any pain after treatment at 6-month follow-up (Table 3).

**Table 3:** Patient satisfaction using VAS scale before and after HADM implantation surgery

	24h after surgery	7 days	1 month	3 months	6 months
VAS	8.61 ± 2.39	5.29 ± 2.37	6.32 ± 1.33	4.05 ± 0.62	1.61 ± 0.31

## Discussion

Vaginal hyperlaxity syndrome (VHS) is a new concept related to excess looseness of the vaginal walls, and rejuvenation techniques are gaining popularity among women to correct this condition [16]. It may develop after pregnancy and vaginal delivery, or by prior pelvic surgery, menopause, and aging [17]. Till date, there is no conclusive definition or diagnostic criteria available for vaginal laxity [18]. In a survey of physician members of the International Urogynecological Association (IUGA), it was revealed that vaginal laxity is under reported by 83% of 563 respondents [19]. Nonrelaxing pelvic floor dysfunction is caused by relaxed muscles and may lead to pelvic organ prolapse or urinary incontinence. This results in pain and problems with defecation, urination, and sexual function adversely affecting quality of life [20]. Therefore, in patients with vaginal constriction, strengthening of pelvic floor muscles is very important to improve vaginal contraction and enhance sexual function.

Surgical methods, namely vaginoplasty and perineoplasty, have been used traditionally for wound repair after childbirth. Recently, these techniques have been increasingly used for vaginal laxity and aesthetic treatment [21]. Fibrillar collagens (collagen types I, II, and III) are the predominant matrix components that play an important role in maintenance of vaginal support by maintaining its tensile strength. Moreover, total collagen is decreased in women with pelvic organ prolapse and hence normal secretory function is not maintained [22]. The goblet cells of vaginal mucosa are reduced, and the mucosal fold wall tends to be flat or even disappear due to aging or childbirth. This vaginal atrophy may result in thinning of the epithelium, the smoothing of mucous folds, reduced blood flow, decreased epithelial glycogen content, reduced lubrication, and dryness thereby affecting sexual life [23]. Thus it can be confirmed from the above literature that vaginal lubrication is essential for sexual arousal, thereby facilitating intercourse. In the present study, it was found that patients complained of vaginal dryness, especially in the early 1–3 months after vaginal constriction surgery. This dryness is attributed to stress mucosal injury caused by surgery, slow recovery of submucous wound and psychological factors impacting the vaginal secretion, leading to dry discomfort. To address this issue, the present technique has been modified to include EPT after HADM implantation to preserve the vaginal mucosa so as to retain good secretion and sensitivity in the early postoperative period.

Our results show that the injection of EPT after HADM implantation could improve the vaginal laxity and sexual function in patients experiencing dryness post-HADM implantation. The VHI score was significantly increased post-3 months of treatment. These improvements were maintained for the 6 months of study. At the same time, there was improvement in the quality of patients’ sex lives after treatment with enhanced vaginal trophicity. Our results were consistent with a study by Hersant et al. [9] which reported that treatment with PRP-HA in postmenopausal women with breast cancer patients improved the trophicity and hydration of vaginal mucosa. The FSD score also significantly increased by 17% in that study. Another retrospective study showed that injecting PRP injections reduced sexual distress among women suffering from vaginal atrophy. The authors speculated that injecting PRP into the affected anatomical areas could improve sexual responsiveness by releasing growth factors and cytokines in the areas near the injection site, thus restoring and improving physiological responsiveness [24]. A case study on women undergoing vulvovaginal rejuvenation procedure reported that injection of PRP-HA improved vaginal trophicity [25].

PRP is rich in a variety of growth factors, such as platelet-derived growth factor (PDGF), transforming growth factor (TGF-B), insulin-like growth factor (IGF), epidermal growth factor (EGF), fibroblast growth factor (FGF), and vascular endothelial growth factor (VEGF) [26]. These growth factors play an extremely important synergistic role in promoting cell proliferation and differentiation, promoting collagen and matrix synthesis, and deposition and tissue formation [27]. Recently, adipose-derived regenerative cells have shown to be beneficial in the treatment of dermatologic conditions. In a primiparous patient, subdermal injection of these cells resolved the recurrent fourchette fissures which was irresponsive to standard medical and surgical therapies [28]. Lately, the evidence suggested a promising efficacy of PRP injection as adjuvant therapy in the surgical repair of recurrent vesicovaginal fistula.[29] HADM is common known biomaterial which is used in various reconstructive surgeries, vaginal repair, pelvic reconstructive surgery [6, 7, 30]. Given their combined effects, EPT after HADM in combination might offer a new alternative of treatment in patients who experience vaginal laxity.

The present study utilized new generation Livsraft EPT device with a unique design of centrifuge tube. This device is highly efficient in preparing large and absolute sterile doses of PRP. High concentrations of platelet plasma EPT are obtained which are 5-10 times more concentrated as compared to conventional centrifugal devices. This enables injecting high concentrations of PRP into the entire vaginal mucosa in one centrifugal cycle [31]. There are limitations to our study that should be recognized. Firstly, the sample size was limited which may introduce an element of bias; hence, research in large population is warranted to further elucidate the feasibility of this new technique. Secondly, we only included subjective scores to assess the efficacy of this technique. Quantification of this technique using invasive or non-invasive methods such as imaging and biopsy will aid in understanding the histological changes, skin elasticity better. Thirdly, there was an absence of comparative control, future studies with an active comparator or placebo will help to strengthen the study findings. Studies evaluating the effect of HADM or EPT alone in future studies are warranted.

## Conclusion

To conclude, EPT injection supplemented the collagen of mucous membrane, thereby increasing the secretory function, and lubrication of vagina. Therefore, HADM implantation followed by EPT was safe and associated with both improved vaginal laxity and sexual function. These results may provide a novel surgical technique for this less recognized and undertreated condition.
